# Intrathyroidal Thymic Tissue in an Adolescent with Graves' Disease: Case Report and Review of Current Literature

**DOI:** 10.1155/2019/8089714

**Published:** 2019-04-01

**Authors:** Liane Eng, Lisa Underland, Leslie Lam

**Affiliations:** Division of Pediatric Endocrinology & Diabetes, The Children's Hospital at Montefiore, 3415 Bainbridge Avenue, Bronx, NY 10467, USA

## Abstract

Intrathyroidal thymic tissue (ITT) is a benign entity found in children and young adolescents that often mimics a concerning thyroid nodule with microcalcifications on ultrasound. It is challenging for the clinician to distinguish between these two entities, which may lead to unnecessary invasive procedures. We report an adolescent female patient with Graves' disease who underwent total thyroidectomy for a thyroid nodule concerning for malignancy for which the surgical pathology ultimately revealed ITT. As ITT is rarely found beyond childhood, the concurrent Graves' disease may have led to persistence of thymic tissue in this patient. Several sonographic features can help in differentiating ITT from a concerning thyroid nodule. Once identified, ITT should be followed by serial imaging with anticipation of decreasing size or complete resolution over time.

## 1. Introduction

Thyroid nodules are uncommon in the pediatric population but have higher rates of malignancy and exhibit higher risk for regional and distant metastases at presentation than in the adult population [1]. It is the eighth most common cancer in adolescents and the second most common cancer in girls [1]. Sonographic characteristics that suggest malignancy include microcalcifications, irregular borders, and hypoechogenicity. Identification of these features on ultrasound prompts the provider to pursue more invasive testing to rule out malignancy. Rarely, ectopic variants of nonthyroidal tissue are identified on ultrasound and may appear similar to a thyroid nodule with concerning features. It is challenging for the provider to differentiate between these benign lesions and a malignant nodule. We present an adolescent patient with Graves' disease who was found to have a thyroid nodule with concerning features on ultrasound for which surgical pathology revealed intrathyroidal thymic tissue (ITT).

## 2. Patient Presentation

The patient is an 18-year-old female with Graves' disease and history of medication nonadherence who presented to the emergency room with fevers, chest pain, palpitations, and diarrhea. She had been diagnosed with Graves' disease two months earlier with an initial presentation of unintentional weight loss, palpitations, tremors, and diarrhea, but no compressive symptoms. She was prescribed methimazole which she intermittently took. Her family history was significant for a cousin with thyroid cancer, mother with uterine cancer, half-brother with skin cancer, and two relatives with breast cancer.

In the emergency room, she had tachycardia (heart rate 155 beats/minute) and orthostatic hypotension. She was afebrile. Her exam was notable for a diffusely enlarged thyroid with no palpable nodules. An electrocardiogram showed sinus tachycardia, and a chest X-ray was negative.

**Figure 1:**
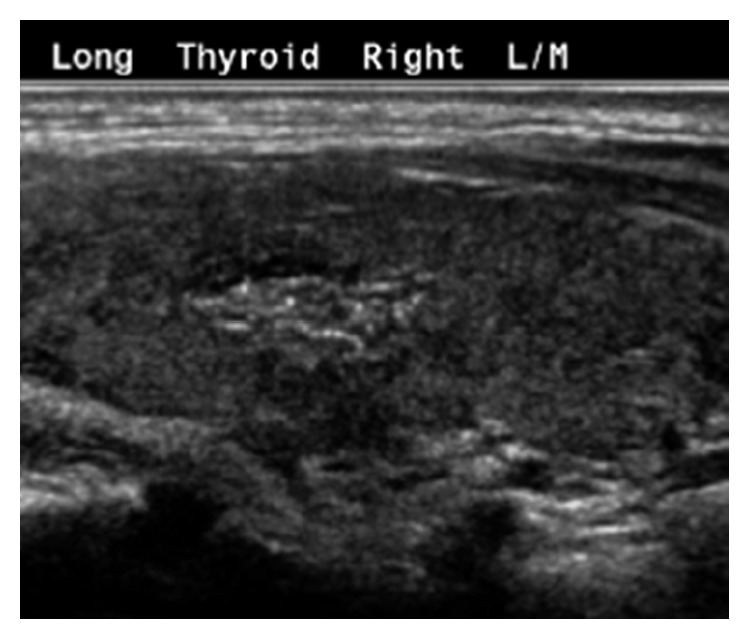
Ultrasound finding showing a 1.3 × 1.7 × 1.1 cm focal area of ill-defined echogenicity in the midright lobe with ill-defined margins, linear echogenic areas, and internal vascularity.

She was admitted due to concern for impending thyroid storm and was started on atenolol and restarted on methimazole. Her symptoms improved gradually after initiation of these medications. During the hospitalization, she had a thyroid ultrasound which showed an enlarged hyperemic thyroid gland consistent with Graves' disease and an indeterminate focal area (1.3 × 1.7 × 1.1 cm) in the right lobe containing abnormal linear echogenicities (Figure 1). She was discharged home on methimazole with outpatient follow-up. At her follow-up visit two weeks later, the patient had a repeat ultrasound which showed a similar 1.7 cm nodule with indistinct margins containing linear and punctate echogenicities, concerning for microcalcifications. A fine-needle biopsy of the nodule was performed, and it showed a benign cluster of reactive follicular cells and lymphocytes (Bethesda II). Despite the cytology results, there was a continued concern for malignancy given the sonographic features of the nodule in the first two ultrasounds, so repeat imaging was performed four months later, which showed minimal change in the nodule size and features.

**Figure 2:**
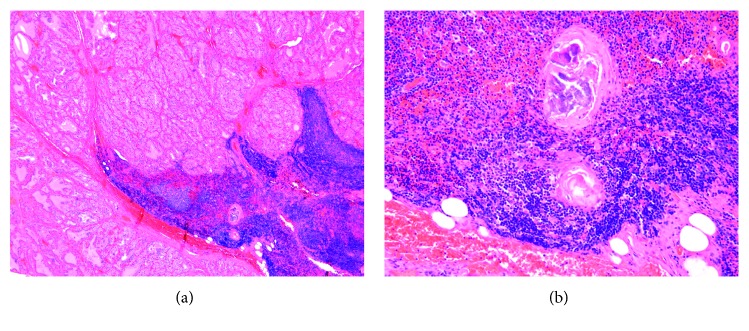
Pathology of the thyroid nodule. (a) Low power view of the thyroid nodule showing focus of lymphocytic predominance, consistent with thymic tissue. (b) High power view of the area of thymic tissue with Hassall's corpuscles, a pathognomonic finding for thymic tissue.

Given the poorly defined thyroid nodule and the patient's desire for definitive therapy for the hyperthyroidism, she underwent a total thyroidectomy. The surgery was complicated by postoperative hypoparathyroidism for which she received intravenous and oral calcium and oral calcitriol. Surgical pathology identified benign thyroid tissue with papillary hyperplasia and a benign nodule of ITT with foci of Hassall's corpuscles with calcifications (Figure 2). At follow-up visits, she was started on levothyroxine for postoperative hypothyroidism. Her calcium levels improved such that calcitriol was discontinued and oral calcium dosing was decreased.

## 3. Discussion

**Figure 3:**
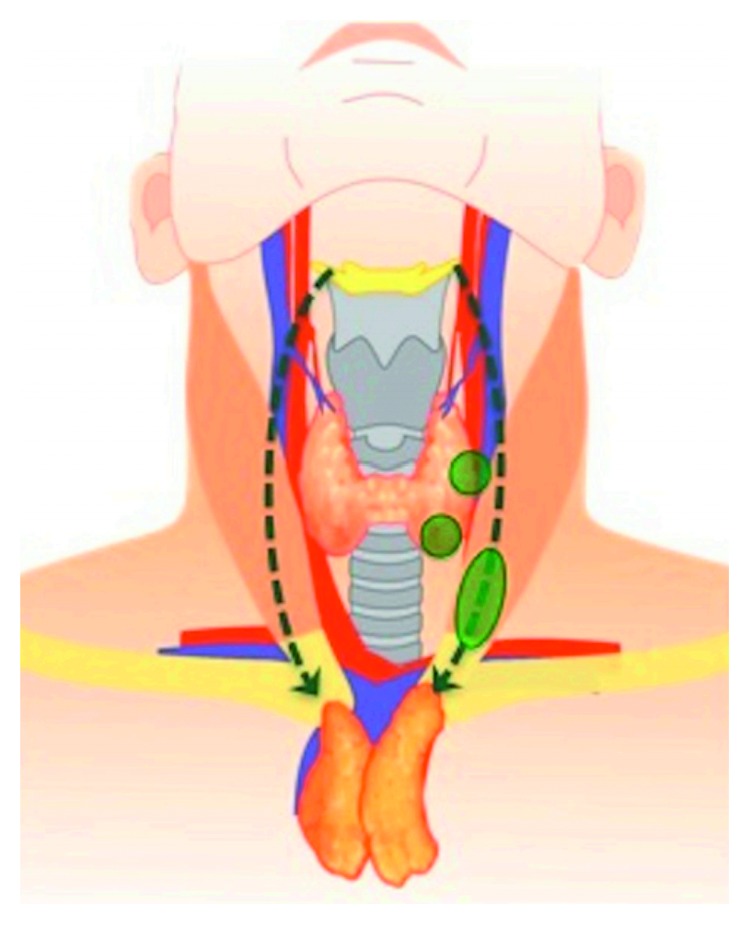
Path of descent for thymic lobes during embryological development (dotted arrow) and locations of ectopic thymic tissue (green ovals) [2].

The thymus arises as two separate lobes from each of the third pharyngeal pouches around the 6th week of gestation during embryonic development. Each lobe descends along a path and fuses in the midline to form the thymic gland by the 8th week of gestation. It may slightly increase in size in early puberty but typically involutes by mid-to-late puberty. Maldescent of thymic tissue during embryogenesis may lead to remnant tissue left along the path of descent, resulting in ectopic thymic tissue (Figure 3) [2]. While ectopic thymic tissue is more commonly seen in the cervical region, it can rarely be found within the thyroid gland.

The sonographic appearance of the thymus or thymic tissue is a hypoechoic lesion which contains punctate or linear echogenic structures, often described as a “starry sky.” When present within the thyroid gland, it is challenging to distinguish this from a concerning thyroid nodule with microcalcifications. Several studies have examined the sonographic features of ITT and have found that they are typically found in the mid-to-lower portions of the thyroid lobes, are fusiform in shape with well-defined margins, and are hypovascular (Table 1) [3–5]. This is in comparison with suspicious thyroid nodules, which tend to be tall in shape, have irregular margins, and are hypervascular. The absence of cervical lymphadenopathy and the comparison of the nodule architecture to the thymic gland, when visible, may further aid in diagnosis.

Segni and his colleagues followed nine patients with ITT over time and found that the tissue either remained stable in size or decreased in size, consistent with the natural course of thymic tissue [7]. Other researchers have also found similar pattern over time, suggesting that this is a benign entity (Table 2) [3–7]. While invasive evaluation, such as fine-needle aspiration and/or surgical removal, is recommended by the current pediatric guidelines for concerning thyroid nodules [1], there is no such indication for ITT as this is a benign entity with no malignancy potential. Instead, it is recommended to perform serial thyroid ultrasounds and concurrently visualize the normal thymus gland in the same patient for comparison. If a fine-needle aspiration is performed, flow cytometry using lymphocyte markers can be used to support the diagnosis of ITT [3].

As thymic tissue typically involutes by puberty, ITT is rarely found in late adolescence and adulthood. ITT was found in our patient at a later age than expected. To the authors' knowledge, there is only one previously reported case of ITT found in a 23-year-old female patient with uncontrolled Graves' disease [8]. In this particular patient, no thyroid nodule was seen on ultrasound, and the ITT was incidentally found postoperatively on pathology [8]. Persistence of the thymic tissue beyond young adolescence in our patient may be related to her concomitant Graves' disease. Thymic hyperplasia is known to be associated with Graves' disease. Though the mechanism is not known, it is hypothesized to be through hormonal and immunological processes [9]. Given this association, the concurrent Graves' disease in our patient may have slowed or halted the anticipated involution of thymic tissue during puberty, leading to its persistence in late adolescence.

Thyroid nodules are typically evaluated as either malignant or benign thyroid tissue. This patient presentation highlights the importance of considering ITT in the differential diagnosis of a thyroid nodule. For a nodule with sonographic features of ITT and no other indication for thyroidectomy, monitoring of the nodule may be appropriate.

## Figures and Tables

**Table 1:** Comparison of the sonographic features of intrathyroidal thymic tissue and concerning thyroid nodule.

Feature	Intrathyroidal thymic tissue	Suspicious thyroid nodule
Side	Left > right	
Location	Midlower portions of lobe	
Shape	Fusiform	Tall shape
	Well-defined margins	Irregular margins
Echo pattern	Hypoechoic with linear/punctate echogenic structures	Hypoechoic with microcalcifications
Vascularity	Hypovascular	Hypervascular
	Isovascular	
Cervical lymph nodes	Normal	Abnormal

**Table 2:** Literature review: patients with follow-up ultrasounds for intrathyroidal thymic tissue.

Study	Number of patients	Interval of follow-up ultrasounds	Changes in intrathyroidal thymic tissue appearance
Segni et al. [7]	9	Mean 34 months	2/9 with reduced size
		(6–84 months)	1/9 with increased size (age 13 months)

Kim et al.[3]	3	6, 11, and 18 months	3/3 with stable size and echo pattern

Yildiz et al. [5]	9	Mean 15.2 months	8/9 with stable size
		(0–48 months)	1/9 with reduced size

Vlachopapadopoulou et al. [4]	36	6, 12, and 18 months	31/36 with stable size
			4/36 with reduced size (adolescents)
			1/36 with resolution (1.5 years later)

Frates et al. [6]	8	2 months—5 years	7/8 with stable size
			1/8 with resolution (5 years later)
